# A Comparison of Methodological Approaches to Measuring Cycling Mechanical Efficiency

**DOI:** 10.1186/s40798-019-0196-x

**Published:** 2019-06-10

**Authors:** Pekka Matomäki, Vesa Linnamo, Heikki Kyröläinen

**Affiliations:** Faculty of Sport and Health Sciences, Biology of Physical Activity, University of Jyväskylä, Jyväskylä, Finland

**Keywords:** Gross efficiency, Delta efficiency, Work efficiency, Delta efficiency, Economy, Energy expenditure

## Abstract

**Background:**

Much is known about theoretical bases of different mechanical efficiency indices and effects of physiological and biomechanical factors to them. However, there are only a few studies available about practical bases and interactions between these efficiency indices, which were the aims of the present study.

**Methods:**

Fourteen physically active men (*n* = 12) and women (*n* = 2) participated in this study. From the incremental test, six different mechanical efficiency indices were calculated for cycling work: gross (GE) and net (NE) efficiencies, two work efficiencies (WE), and economy (*T*) at 150 W, and in addition delta efficiency (DE) using 3–5 observation points.

**Results:**

It was found that the efficiency indices can be divided into three groups by Spearman’s rank correlation: GE, T, and NE in group I; DE and extrapolated WE in group II; and measured WE in group III. Furthermore, group II appeared to have poor reliability due to its dependence on a work-expended energy regression line, which accuracy is poorly measured by confidence interval.

**Conclusion:**

As efficiency indices fall naturally into three classes that do not interact with each other, it means that they measure fundamentally different aspects of mechanical efficiency. Based on problems and imprecisions with other efficiency indices, GE, or group I, seems to be the best indicator for mechanical efficiency because of its consistency and unambiguity. Based on this methodological analysis, the baseline subtractions in efficiency indices are not encouraged.

## Key Points

- Efficiency indices fall naturally into three classes that do not interact well with each other.
- Because of its consistency and unambiguity, gross efficiency seems to be the best indicator for mechanical efficiency. There seems to be both unsolved methodological as well as theoretical problems and imprecisions with other efficiency indices.
- The baseline subtractions in efficiency indices are not encouraged.

## Background

There are at least three reasons to study mechanical efficiency and economy of a locomotion: (1) theoretically, they are considered as important components to explain endurance performance in general [1, 2], although, particularly in cycling, their importance might not be so strong [3, 4]; (2) knowing how different factors, such as temperature [5] and fatigue [6], affect efficiency and economy can give valuable information about how muscles and body act; and (3) theoretical interest of knowing how efficient an isolated musculoskeletal system can be [7, 8]. Partly because of this last reason, it is a common practice to try to subtract all energy costs that are not directly related to a work production and try to isolate components primarily responsible to the movement to reach the mechanical efficiency of the isolated moving muscles. This has inspired to define many slightly varying indicators for the mechanical efficiency. In this study, six most utilized metabolism based indicators from literature were chosen. Theoretical consideration and pros and cons together with a success of them to describe the efficiency of an isolated musculoskeletal system have widely been discussed and analyzed [6–13]. To get an overview of these different efficiency indices, what follows is a short presentation of these mechanical efficiency indices (see also Table 1).

**Table 1:** Mechanical efficiency indices utilized in the present study and their interpretations

Indicator	Interpretation	Definition
GE	Mechanical efficiency of a whole body	$\frac{W_{\mathrm{ext}}}{E_{\mathrm{tot}}}$
*T*	Rough indicator for GE	$\frac{{\mathrm{VO}}_2}{P_{\mathrm{out}}}$
NE	Mechanical efficiency for everything that can have adaptations	$\frac{W_{\mathrm{ext}}}{E_{\mathrm{tot}}-{E}_{\mathrm{rest}}}$
WE	Mechanical efficiency of an isolated musculoskeletal system	$\frac{W_{\mathrm{ext}}}{E_{\mathrm{tot}}-{E}_0}$
DE	Averaged mechanical efficiency of an isolated musculoskeletal system	$\frac{{\Delta W}_{\mathrm{ext}}}{{\Delta E}_{\mathrm{tot}}}$

One of the most widely used indicator for mechanical efficiency is gross efficiency $\mathrm{GE}=\frac{W_{\mathrm{ext}}}{E_{\mathrm{tot}}}$, where *W*_ext_ is the accomplished external work and *E*_tot_ the total energy expenditure. A very closely related index is economy $T=\frac{{\mathrm{VO}}_2}{P_{\mathrm{out}}}$, where VO_2_ is the oxygen consumption and *P*_out_ is the power output. Economy can be interpreted as a rough alternative for GE, as it does not take carbon dioxide (VCO_2_) production into account. Of all indicators, GE is the simplest one, which is its major strength. Unlike the other efficiency indices, its calculation and interpretation are simple and unequivocal: GE is a mechanical efficiency of a whole body in a cycling work.

In the history of efficiency, the aim has been to attain an isolated efficiency of the musculoskeletal system. One way to approach this would be to make a right baseline subtraction for *E*_tot_. Net efficiency $\mathrm{NE}=\frac{W_{\mathrm{ext}}}{E_{\mathrm{tot}}-{E}_{\mathrm{rest}}},$ where *E*_rest_ (resting energy expenditure) is the chosen baseline subtraction and is the simplest attempt into this direction. However, NE does not take into consideration the fact that the energy expenditure of supporting homeostasis increases as the external resistance increases [10]. On the other hand, to show the ambiguous nature of interpreting NE, it can be argued that NE does not even try to describe the effectiveness of the musculoskeletal system, but instead how well individual has adapted to a cycling movement. In principle, as one cycle an ergometer, all energy expenditure above *E*_rest_ is caused by the cycling work, in a way or another, being thus susceptible to adaptations.

The next step towards efficiency of the musculoskeletal system is work efficiency (WE). Here the baseline subtraction is *E*_0_, an energy expenditure of a pedaled zero load, which tries to capture an internal energy expenditure of a cycling work. On top of *E*_rest_, *E*_0_ also captures energy expenditure for moving lower body segments and for the slightly increased metabolism requirements due to this movement [11]. However, there are both theoretical and practical difficulties. On the theoretical side, *E*_0_ does not take into consideration the fact that internal energy expenditure increases as pedaled resistance increase [14], and it is quite dubious to assume that the energy expenditure can be divided into independent external and internal components [8, 15]. On the practical side, *E*_0_ can be measured in two ways: either measuring directly the energy expenditure when cycling zero load (*E*_0,*m*_) or extrapolating it from *W*_ext_-*E*_tot_ regression line (*E*_0,*e*_), leading to two different WE, a measured one, $\mathrm{W}{\mathrm{E}}_m=\frac{W_{\mathrm{ext}}}{E_{\mathrm{tot}}-{E}_{0,m}}$, and an extrapolated one, $\mathrm{W}{\mathrm{E}}_e=\frac{W_{\mathrm{ext}}}{E_{\mathrm{tot}}-{E}_{0,e}}$, problem being that *E*_0,*m*_ is vastly greater than *E*_0,*e*_ difference ranging from 20% [16] to 350% [17]. Theoretical reasons for this difference are problems for creating true zero load, body stabilization problem, abnormal cycling situation, and a different muscle activation pattern [16], and as a result, theoretically *E*_0,e_ is favored as true *E*_0_ over *E*_0,m_.

The last easily calculated attempt towards an efficiency of an isolated musculoskeletal system is a delta efficiency $\mathrm{DE}=\frac{{\Delta W}_{\mathrm{ext}}}{{\Delta E}_{\mathrm{tot}}}$, where Δ*W*_ext_ is the change in external work and Δ*E*_tot_ the change in total energy expenditure. In practice, DE is usually calculated as an inverse of a slope from *W*_ext_-*E*_tot_ regression line, and hence, DE can be interpreted as an averaged mechanical efficiency of the musculoskeletal system. DE describes how much one needs to increase energy expenditure from a present state to keep on with the increased work intensity, and in this way, it tries to avoid problems caused by constant baseline subtractions. At least three critical points against DE have been presented. First, it implicitly assumes that the increase in energy expenditure stays constant and is independent from the energy expenditure of a present state [8]. Second, DE is based on a linearity of *W*_ext_-*E*_tot_ regression line, which is violated, e.g., by the slow VO_2_ component, which may be visible already when intensity exceeds ~ 50% VO_2max_ [18, 19]. Third, the repeatability of DE is, for unexplained reason, quite weak [10]. The interpretations and definitions of different efficiency indices are gathered in Table 1. Usually, the soundest efficiencies have theoretically been argued to be either GE [6, 8, 20] or DE [7, 9, 21].

In the present study, we only consider the above mentioned metabolically defined efficiency indices as they form a coherent entity. An alternative efficiency indicator involving internal mechanical work has also been introduced: $\frac{W_{\mathrm{int}}+{W}_{\mathrm{ext}}}{E_{\mathrm{tot}}-{E}_{\mathrm{rest}}}$ [22–24]. In it, the summation of internal and external works has faced criticism, the main one being that the internal and external works overlap in such a way that one has a risk to count twice a part of the work done [8].

It should be mentioned that a harsh criticism towards baseline subtractions, in general, have been proposed [8, 12, 13, 25]. One of the main arguments is that the energy representing a baseline is changing when work rate changes. As is discussed in [25], the increasing work rate affects the body. For example, it increases the overall metabolic rate by increasing mean body temperature and inducing hormonal changes and it also increases the splanchnic metabolism. Another often repeated argument against baseline subtractions is that the energy expenditure cannot be divided into non-overlapping components. Despite the criticism against baseline subtractions, they are quite widely used.

Nowadays, much is known about the effects of different physiological and biomechanical factors to these differently defined indicators of mechanical efficiency [5, 8, 9, 26–33]. However, it is less studied how these differently defined indicators of mechanical efficiency interact with each other and how sensible these indices are methodologically. In the present study, we made a hypothesis that if all the different indicators of mechanical efficiency would measure principally the same feature, a cyclist who is good with regard to one efficiency indicator is good also with all the other indicators. We were also interested in the accuracy of *W*_ext_-*E*_tot_ regression line, as DE and WE_*e*_ are calculated from it.

## Methods

### Subjects

The study included 14 subjects (12 males and 2 females) from different sport backgrounds, and they were sport science students or members of local sport clubs. Practically, all of them used cycling as their training mode in some part of the year. Six of them could be classified as cyclists (i.e., active cyclist or triathlete). The intensity of 150 W was chosen to be the power output at which the different efficiency indices were compared. This was thought to be intensity high enough so that the internal energy expenditure does not interfere with the outcome. To make sure that this load of 150 W was completed mostly aerobically, a candidate was accepted to the study only if the following criteria were fulfilled: respiratory exchange ratio (RER) at 150 W was not more than 1.00 and the aerobic threshold was at least 150 W, determined by Finnish standards based on the first clear increase of lactate level from baseline [34]. By the terminology of Seiler [35], these criteria ensure that 150 W was in a light intensity training zone, making it a relatively easy load for each participant. Two educated testers defined independently aerobic thresholds for the participants. Of the 17 tested subjects, 14 participants (12 men and 2 women) fulfilled the inclusion criteria. For them, the mean (± SD) increase in lactate at 150 W load from its lowest value was 0.38 **±** 0.29 mmol/l. Their further information is given in Table 2.

**Table 2:** The basic information of the subjects

	Age (years)	Height (cm)	Body mass (kg)	VO_2max_ (ml/kg/min)	*P*_max_ (*W*)
Mean ± SD	31 ± 6	180 ± 6	75.8 ± 10.6	53.3 ± 6.0	325 ± 46
Range	21–39	170–195	65.0–105.1	42.7–64.6	250–420

*VO*_*2max*_ maximal oxygen consumption, *P*_*max*_ maximum power in incremental test

### Experimental Design

Each subject was taken into a test room separately, and the temperature (20–22 °C) and humidity (25–30%) were quite stable during and between each test. Subjects were asked to refrain from strenuous exercise 2 days prior to a test. First, the body height and weight were measured. The weight was measured with cycling clothes without shoes and, in addition, 300 g was subtracted from this as weight for the clothes and to anticipate the weight reduction due to perspiration.

The ergometer test began with 10-min resting gas exchange measurement (MasterScreen CPX, CareFusion, San Diego, USA) by sitting still on the cycling ergometer (Monark Ergomedic 839E), which is a customary way to measure *E*_rest_ in efficiency literature [7, 32]. After this, to measure *E*_0,*m*_, the participant cycled 5 min against zero load. The initial load for the incremental test was chosen between 90 and 150 W depending on the fitness level of the participant, but in such a way that each subject performed the demanded 150 W. The load was increased by 30 W (men) or 25 W (women) at every 5 min. The gas exchange was measured by breath-by-breath method and analyzed with Lab Manager V5.32.0 (Laboratory Systems Group Pty. Ltd., Melbourne, Australia) with the average of last minute, except with *E*_rest_ two last minutes, of each load was taken into analyses. Lactate was taken from the fingertip immediately after each load, and it was analyzed by Biosen C_line Sport 2 (EKF Diagnostic lactate/glucose, Cardiff, UK). The test was progressed in this fashion until the lactate was increased by 1 mmol/l from the initial level, after which, to measure VO_2max_, the load was increased every 2 min until voluntary exhaustion or until cadence reduced irrevocably (> 15 s) below 60 rpm. During these final loads, the participants were strongly encouraged. Participants were free to choose their favored cadence (the realized range was 70–95 rpm), but it was instructed to keep constant during the 5-min loads, as cadence affects the efficiency [28]. A metronome was offered for maintaining a constant cadence. Moreover, the test conductors kept watching over the cadence inspecting visually the cadence meter. Further, during the 5-min loads, the participants were instructed to stay seated and keep their hand on the same spot on the handlebar, as the position on the bike may affect efficiency [8]. The cadence, shoes, and riding position were freely chosen by the participants, as we were interested in subjects’ efficiency indices as they appear in their regular cycling practices.

### Processing Data

Aerobic energy expenditure was calculated by applying the equation from [34]:

$$ {E}_{\mathrm{tot},\mathrm{Aer}}\left(\mathrm{kJ}/\min \right)=\left(5.05\ \left(\mathrm{kJ}/\mathrm{l}\right)\times \mathrm{RER}+16.1\ \left(\mathrm{kJ}/\mathrm{l}\right)\right)\times {\mathrm{VO}}_2\ \left(\mathrm{l}/\min \right). $$

Furthermore, lactate measurements were utilized to estimate the anaerobic energy expenditure during the 5-min loads by applying the formula from [36]:

$$ {E}_{\mathrm{tot},\mathrm{Anaer}}\left(\mathrm{kJ}/\min \right)=0.003\ \mathrm{l}/\mathrm{kg}\times \mathrm{body}\ \mathrm{mass}\ \left(\mathrm{kg}\right)\times 21.15\ \mathrm{kJ}/\mathrm{l}/\min \times \frac{\Delta \mathrm{La}\left(\mathrm{mmol}/\mathrm{l}\right)}{5}. $$

Here, ΔLa is the difference of the lactate concentrations between the observed and the previous load. The total energy expenditure was the summation of these two components: *E*_tot_ = *E*_tot,Aer_ + *E*_tot,Anaer_. For each subject, an individual regression line *E*_tot_ = *aW*_ext_ + *b* was calculated by using observation points from the first load up to the aerobic threshold. This led to 3–5 observation points ranged between 90 and 210 W to be used for the regression line, which is quite the typical amount of points in literature [37–39].

### Efficiencies Approach Theoretically DE

It is quite customary to verbally argue how the role of internal energy expenditure *E*_0_ comes negligible when external work *W*_ext_ increases arbitrarily large [8]. Next, we show how this can be calculated strictly and how the theoretical consequence is that every efficiency indices approach to DE. In other words, it is shown mathematically that regardless of their definitions and starting points, every considered mechanical efficiency indices unite as external work rate increases.

The following is the standing assumption.

**Assumption 1** Assume that *W*_ext_-*E*_tot_ function is linear, i.e., that *E*_tot_ = *aW*_ext_ + *b*, for some *a*, *b* ∈ *R*_+_.

By definition, DE is an inverse of a slope of *W*_ext_-*E*_tot_ regression line, which is linear by Assumption 1. That is $\mathrm{DE}=\frac{1}{a}$, where *E*_tot_ = *aW*_ext_ + *b*. Furthermore, by definition $\mathrm{GE}=\frac{W_{\mathrm{ext}}}{E_{\mathrm{tot}}}$, so that when applying Assumption 1, one can derive

$$ \mathrm{GE}=\frac{W_{\mathrm{ext}}}{E_{\mathrm{tot}}}=\frac{W_{\mathrm{ext}}}{a{W}_{\mathrm{ext}}+b}=\frac{1}{a+\frac{b}{W_{\mathrm{ext}}}}. $$

Here, the last expression approaches to $\frac{1}{a}=\mathrm{DE}$ when *W*_ext_ approaches to infinity, as $\frac{b}{W_{\mathrm{ext}}}$ approaches to zero. This gives and proves that GE approaches to DE as *W*_ext_ approaches to infinity. Using similar deduction, it can also be concluded that NE and WE approach to DE as *W*_ext_ approaches to infinity.

### Statistical Analysis

The relations between mechanical efficiency indices were studied applying Spearman’s rank correlation. Normal distribution of the variables, when needed, was tested using Shapiro-Wilk test. Lastly, the difference between the groups was tested by *t* test. All statistical tests were performed as two-sided with statistical significance level set at 0.05. Values have been reported as mean ± SD. The analyses were done applying Apache Open Office (Open Office 4, Apache Foundation, USA), MS-Excel (Office 2013, Microsoft, USA), and Mathematica 7 for Windows (Wolfram Research, USA).

## Results

### Efficiencies

The following Table 3 contains the measured efficiency index values. At 150 W, the measured *E*_tot,Anaer_ was 0.25 ± 0.18 kJ/min, while *E*_tot,Aer_ was 44.8 ± 1.8 kJ/min.

**Table 3:** Mean (± SD) values for the measured efficiency indices

Efficiency	GE (%)	*T* (ml/min/W)	NE (%)	DE (%)	WE_*e*_ (%)	WE_*m*_ (%)
Mean (± SD)	20.0 ± 0.8	14.3 ± 0.6	23.4 ± 1.0	23.8 ± 1.9	23.8 ± 1.9	32.0 ± 2.9
Range	18.4–21.4	13.4–15.6	21.5–25.1	20.9–27.3	20.8–27.4	28.0–38.3

*GE* gross efficiency, *T* economy, *NE* net efficiency, *DE* delta efficiency, *WE*_*e*_ work efficiency with extrapolated zero load energy expenditure, *WE*_*m*_ work efficiency with measured zero load energy expenditure

### Relationships Between Efficiencies

**Fig. 1:**
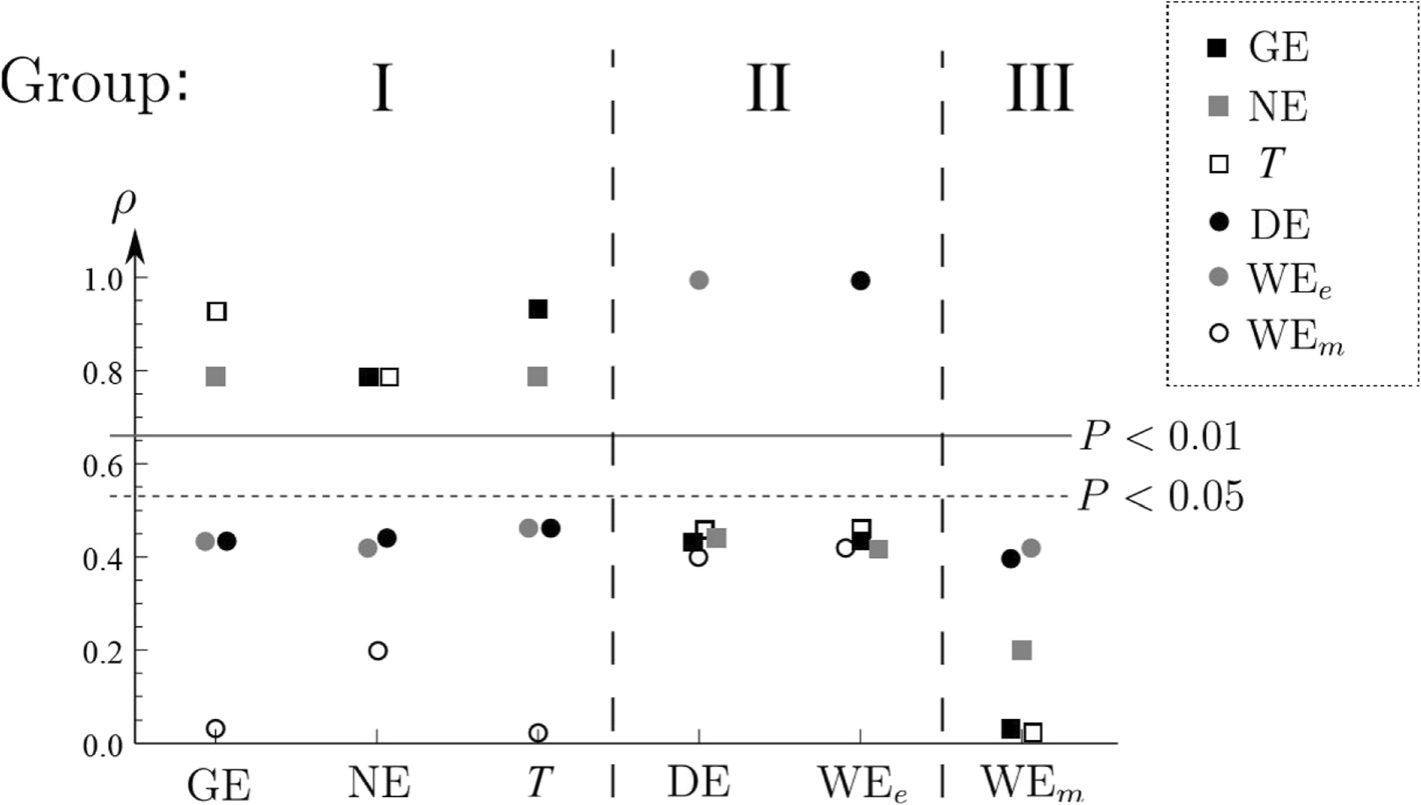
Spearman’s rank correlations *ρ* for the inspected six indices of mechanical efficiency. With two transversal lines, values corresponding to significance levels *p* < 0.05 and *p* < 0.01 have been drawn. The indices can be divided into three groups: groups I, II, and III. GE gross efficiency, NE net efficiency, *T* economy, DE delta efficiency, WE_*e*_ work efficiency with extrapolated zero load energy expenditure, WE_*m*_ work efficiency with measured zero load energy expenditure

The efficiency indices can be divided into three groups by Spearman’s rank correlation (Fig. 1): I (GE, NE, *T*), II (DE, WE_*e*_) and III (WE_*m*_). Grouping was done by selecting two efficiencies into the same group, if their rank correlation exceeded 0.66 (limit for *p* < 0.01 significance). None of the six indicators of efficiency belonged to a more than one group, so that the groups did not overlap with each other. In other words, correlations within a group are strongly significant (group I, *p* ranges between 1×10^−6^ – 0.0008; group II, *p* = 10^−13^), while there are no significant correlations between groups (see Fig. 1).

### Distance Between GE and DE

In the present study, the GE and DE were not very close to each other: the shortest distance between GE and DE were 3.4± 1.7% points, the range being 0.7–7.2% points. It should be mentioned that 10/14 participants had maximal GE value at a higher intensity level than 150 W. Furthermore, for each subject, a regression line *E*_tot_ = *aW*_ext_ + *b* can be constructed. This can be substituted into the ratio $\mathrm{GE}=\frac{W_{\mathrm{ext}}}{E_{\mathrm{tot}}}$ in order to estimate in which theoretical intensities GE would be near DE, if one could pedal infinitely high intensities aerobically. In this study, GE would be theoretically in a 0.5% point environment of DE at intensities of 1350 ± 750 W.

### Energy Expenditure at Zero Load and at Rest

**Fig. 2:**
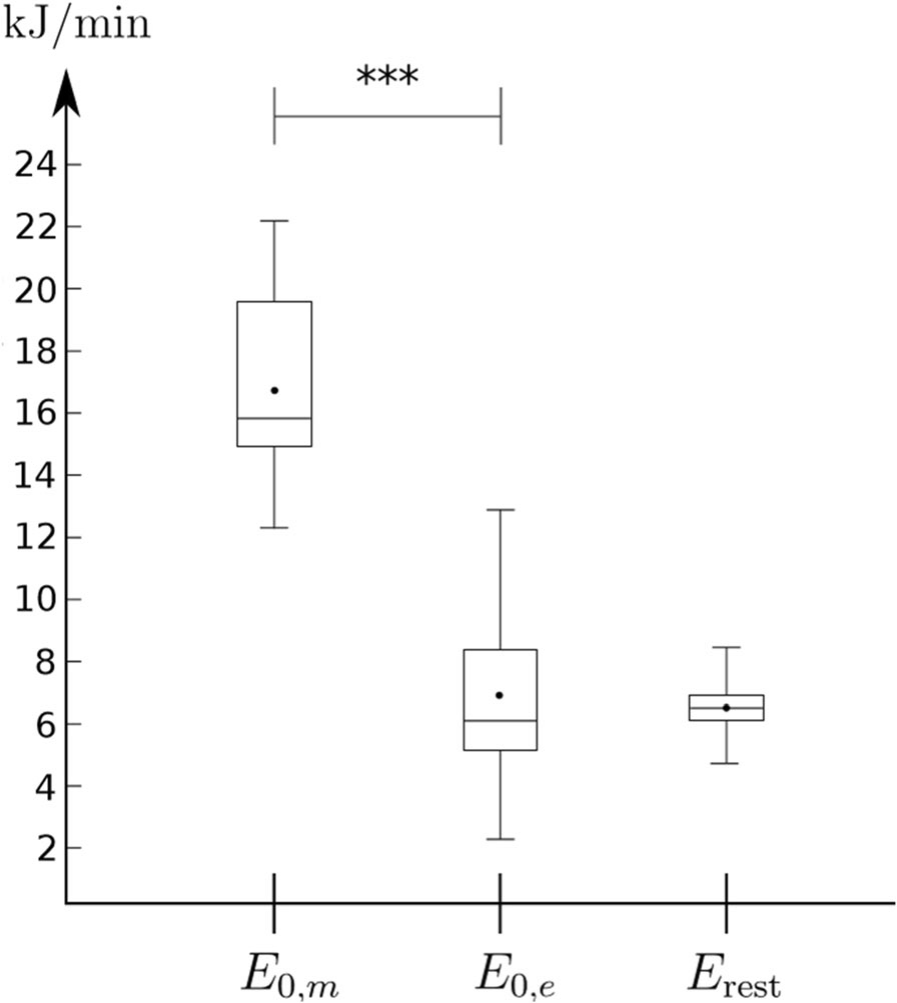
Boxplot for resting energy expenditure (*E*_rest_) and measured (*E*_0, *m*_) and extrapolated (*E*_0, *e*_) zero load energy expenditure. ***Significance with 0.0001 level

Energy expenditures *E*_0,*m*_, *E*_0,*e*_, and *E*_rest_ are plotted in Fig. 2. Measured zero load energy expenditure *E*_0,*m*_ was on average 142% greater than extrapolated *E*_0,*e*_, and they differed from each other significantly (*p* = 4 × 10^−9^). Furthermore, *E*_rest_ did not differ significantly from *E*_0,*e*_ (*p* = 0.60). *E*_0,*e*_ and *E*_0,*m*_ correlated only weakly (*r* = 0.45, *p* = 0.11). Lastly, to estimate a reliability and accuracy of DE and WE_e_, the length of subjects’ 95% confidence interval (CI) for DE and *E*_0,*e*_ were calculated and they were 6.9 ± 5.2% and 47± 32 kJ/min, respectively, while the mean values for DE and *E*_0,*e*_ were 22.6% and 6.9 kJ/min, respectively.

## Discussion

The main result of the present study was that the six investigated indicators for mechanical efficiency formed three separated groups by rank correlation: the first group (group I) was formed by GE, NE, and *T*; the second one (group II) by DE and WE_*e*_; and the third one (group III) by WE_*m*_. Identical grouping would be achieved also by Somers’ D function (not shown). There were strong correlations within the groups, whereas correlations between the groups were at most moderate. As the six indicators of mechanical efficiency fall into different groups, they can be interpreted to measure fundamentally different aspects of mechanical efficiency.

One speculative reason for the observed grouping might be that the baseline subtraction is altogether an erroneous way to approach efficiency: in this paper, it is shown that *E*_0,*e*_ does not differ from *E*_rest_, that the confidence interval for DE is too large to be reliable, and that *WE*_m_ provides too large values for work efficiency. These facts give rise to a question of whether DE and WE measure what they supposedly should measure. Previously, there has been mostly theoretical criticism against NE, DE, and WE [8, 12, 13, 25], but the present study is one of the rarely seen methodological study to address this question.

In theory, as shown in the “Methods” section, every indicator of mechanical efficiency approaches DE if pedaled external work could increase unbounded, as the role of internal energy expenditure *E*_0_ comes negligible compared to total energy expenditure *E*_tot_. That the efficiency indices did form different groups was because one cannot pedal large enough intensities aerobically; theoretically, DE and GE are not near each other (0.5% point) until at 1350 W intensity. This is somewhat contrast to the case study of world-class champion in [40], where the GE and DE were found to be within 0.1% point distance already at 300–400 W power output. Based on the estimations of the present study, this kind of unity between GE and DE at so low power level is highly exceptional.

The measured efficiency indices of the present study seem to be in line with the literature. From a review article [8] in literature, GE has mostly been around 18–20% at 150 W ([8], Fig. 2), and mean ±SD for DE from 14 studies was 23.8± 2.6% ([8], Table 1). These values are well in line with the present study with GE =20.0 ± 0.8% and DE=23.8 ± 1.9%.

### Efficiency Groups

In general, group I can be interpreted to illustrate the mechanical efficiency of a whole body in a cycling work. GE and *T* belong to the same group, understandably, as the former one is a refined version of the latter one. The fact that NE belongs to this group indicates that there are no great differences nor adaptations in resting energy expenditure between individuals. On the other hand, in theory, groups II and III try to grab the efficiency of an isolated musculoskeletal system in a cycling work by subtracting, in a one form or another, zero load energy expenditure from the examination. Hence, it seems that *E*_0_ plays a role, and a bigger one than *E*_rest_, when trying to explain why efficiency indices fall into different groups. The importance of *E*_0_ is well in line with a previous study [41], in which it was argued that differences in zero load cycling between individuals explain some of the observed variation in GE between individuals. Another possible reason for the differences between the groups lies in the difficulty and uncertainty of determining WE_*e*_ and DE from *W*_ext_-*E*_tot_ regression line.

DE and WE_*e*_ belong to the same group as they are both calculated from the same *W*_ext_-*E*_tot_ regression line. Furthermore, at each observation point, WE_*e*_ can be seen as an inverse of a slope of a line through that observation point and *E*_0,*e*_, so that WE_*e*_ can be interpreted, more or less, as a local delta efficiency. If all the observation points would fall on the same straight line, WE_*e*_ and DE would coincide.

One can find indirect support from literature to this grouping. When studying correlations between different physiological factors to indicators of mechanical efficiency, it has been demonstrated that physiological factors affect differently to indicators from different groups. For example, a measure from group I has been reported to be significantly affected while the measure from group II has not, e.g., by the temperature of the skin [32], VO_2max_ [10], and body weight [26]. In addition, group I has been reported to be affected while group III has not, e.g., by training [41]. Thus, the literature shows that, based on correlation to physiological factors, there seem to be some groupings for mechanical efficiency indices supporting indirectly our grouping.

### Accuracy of *W*_ext_-*E*_tot_ Regression Line and DE

It has been observed that the repeatability of DE is significantly weaker than GE [10], but this phenomenon has eluded explanations. Here, we argue that this phenomenon can be explained by the weak accuracy of *W*_ext_-*E*_tot_ regression line, which is caused mainly by using too few observation points, typically 3 [37, 38], 4 [39], or at most 6 [32, 42]. In the present study, we replicated the usual way to calculate efficiency indices, which was the reason to include only 3–5 points to our *W*_ext_-*E*_tot_ regression line. As the value (95% CI) for DE was 22.6% (19.2–26.1%) and for *E*_0,*e*_ 6.9 kJ/min (− 16.6–30.4 kJ/min), it is plainly clear that more observation points would be required for a reliable *W*_ext_-*E*_tot_ regression line, and hence, reliable DE and *E*_0,*e*_ estimates. Noteworthy, the coefficient of determination, *R*^2^, is unable to distinguish this problem, as *R*^2^ value for *W*_ext_-*E*_tot_ regression line in our study was 0.996± 0.004. It means that *R*^2^ is far from a sufficient test for explaining the accuracy of *W*_ext_-*E*_tot_ regression line when there are too few observation points; after all, *R*^2^ with two observation points is always 1.00, although this kind of estimation contains huge potential error. Another factor, besides the number of observation points, affecting reliability of *W*_ext_-*E*_tot_ regression line is cadence. It affects energy expenditure, and applying linear regression from [8] we can, purely theoretically, estimate that using four observation points DE can change as much as 1.1 %-points by only altering cadence from 80 by ±1 rpm. As keeping cadence closer than ± 1 rpm to a target cadence during the test is very challenging, it becomes clear that there is quite large built-in imprecision potential in DE measurements. In contrast, keeping cadence 80 ± 1 rpm affects theoretically GE only by 0.1% points.

A clear proposal to improve the accuracy of *W*_ext_-*E*_tot_ regression line would be to use more data points. For example, in the study of Medbø et al. [43], it has been suggested to use at least 10 observation points when estimating *W*_ext_-*E*_tot_ regression line. Another way to improve the estimation would be to include only aerobic intensities. For example, some efficiency studies have included 270 W loads for women [42] and 300 W loads for men [32] when calculating DE. However, without measuring blood lactates, the amount of anaerobic energy expenditure cannot accurately be estimated for these intensities. Not to mention about the potential impact of slow VO_2_ component, which can be present already when the intensity exceeds 50% VO_2max_ [18, 19] skewing the linearity of *W*_ext_-*E*_tot_ relation. Last proposal to get more precise DE would be to monitor accurately the used cadence.

It should be clear in mind that the accuracy of *W*_ext_-*E*_tot_ regression line has more profound meaning than only that of determining DE and *E*_0,*e*_ as it is also used, e.g., to extrapolate theoretical energy (or oxygen) consumption at high-intensity works [43, 44].

### WE_*e*_ vs. WE_*m*_

It has been widely recognized how *E*_0,*m*_ is much greater than *E*_0,*e*_, the difference ranging from 20 to 350% [16, 17], being 140% in the present study. This means that they both cannot accurately describe *E*_0_ which they supposedly illustrate. Above, we have argued how the accuracy of *E*_0,*e*_ is quite weak based on CI. Another, often ignored, charge against *E*_0,*e*_ is that it does not differ from *E*_rest_ (*p* = 0.60, Fig. 2), with half of the subjects in the present study having smaller *E*_0,*e*_ than *E*_rest_, which sounds abnormal. Similar values can be seen, e.g., in [27]. One explanation might be that *E*_0,*e*_ does not actually illustrate the energy expenditure which it has been thought to illustrate. For example, it has been observed in [15] that internal work is neither constant nor independent from external work. This can be interpreted so that, although *W*_ext_-*E*_tot_ connection would be linear, the energy expenditure of a zero load is not found at the intersection with *y*-axis, as we do not know how the internal work is related to the total energy expenditure at different loads. One could also try to explain the possibility of *E*_rest_ to be truly higher than *E*_0_, as starting an exercise against zero load could in principal increase the work load of the heart and legs but at the same time reduce even more the work load of other parts of the body, e.g., the digestive system and internal organs, but this is highly speculative.

On the other hand, also *E*_0,*m*_ has many problems. Firstly, there are all the theoretical explanations, shown in the “Background” section, how *E*_0,*e*_ offers better approximation for *E*_0_ than *E*_0, *m*_. Moreover, in literature, *E*_0,*m*_ (and thus WE_*m*_) has been discarded because of its too high values [7, 8]. An isolated muscle has theoretically been discussed to have mechanical efficiency at most 30% [12, 13]. In the present study, WE_*m*_ was 32.0± 2.9% (range 28.0–38.3%) and hence, too high for a mechanical efficiency of an isolated musculoskeletal system in a cycling work where the usage of elastic energy is minimal [45]. All in all, *E*_0,*e*_ seems too small to be true energy expenditure for zero load and *E*_0,*m*_ too large, and hence, both of them (and thus WE_*e*_ and WE_*m*_) seem to contain unanswered methodological problems. More specifically, as was reported above, WE_*e*_ and DE are quite parallel measurements for a mechanical efficiency, and as such, if the problems related to WE_*e*_ cannot be solved, it casts doubts also on DE, even though its theoretical base would otherwise be firm enough.

### Methodological Doubts on Baseline Subtractions

To bring the discussion to a conclusion, we have now seen how methodologically DE, WE_*e*_, and WE_*m*_ all contain problems casting some serious doubts on sensibility of baseline subtractions. The previous doubts against NE, WE, and DE are essentially theoretical considerations based on the facts that energy expenditure cannot be divided into separated components and that the baseline subtractions are not invariant with different work intensities [8, 12, 13, 25]. The doubts of the present study are based on the methodological outcomes: essentially that WE_*m*_ is too large, that *E*_0,*e*_ is too similar to *E*_rest_, and that 95% CI of DE and *E*_0,*e*_ are too wide. Based on these findings, NE would be the only methodologically sound mechanical efficiency index with a baseline subtraction. However, both NE and GE belong to the same group I. Thus, one can argue that GE carries basically the same information than NE, but without an additional inconvenience and possible source of error by having to measure *E*_rest_. In this way, the present study suggests methodologically that also the need for NE is questionable.

### Limits of the Study

Although the outcome of our study is quite distinctive with three separated groups for mechanical efficiency indices, some weaknesses could affect this conclusion. Letting each participant choose their own natural cadence could have influenced the outcome, as cadence is known to affect the efficiency indices [8]. Although we acknowledged this, the same cadence was not chosen to impose for everyone, as we were interested in individual differences and dividing individuals into different classes based on their natural cycling patterns. We felt that imposing an unnatural cadence to subjects could interfere with that aim. It should be also mentioned that we did not record cadence from pedal revolution to another, which means there might be a small load to load sway in cadence for each participant. This deviation then mostly affects *W*_ext_-*E*_tot_ regression line, and hence, values of DE and WE_*e*_.

In this study, both male and female subjects were included, as our main interest was to compare different indices of mechanical efficiency for subjects of broad backgrounds. We acknowledge that there is a mild gender difference in GE, *E*_0,*m*_, and *E*_0,*e*_, but that they can be explained mostly by the difference in lean leg volume [17]. As interindividual variation in GE and *E*_0_ can in general be explained mostly by body mass and especially by leg mass [11, 14, 26], we felt that gender question was not too restricting in our approach: allowing also female subjects to take part, we felt that we mainly expanded our study to include also lighter body masses. It should be mentioned that the results are unaltered when analyzed with men only (data not shown).

## Conclusion

This study suggests that the six most applied indices for mechanical efficiency in a cycling work can be divided into three groups by rank correlation. In practice, this means that the groups measure fundamentally different aspects of mechanical efficiency and that results concerning efficiency indicator from one group cannot be straightforwardly adopted to indicators from other groups. This study also shows how the present custom to determine the *W*_ext_-*E*_tot_ regression line seems to be inadequate mostly because of using too few observation points, explaining, at least partly, the repeatable problem of DE.

To conclude, based on methodological problems and imprecisions with other efficiency indices, it seems that GE, or more generally group I, would be the best indicator for mechanical efficiency because of its consistency and unambiguity. Moreover, the use of baseline subtractions is not encouraged. This is in line with the suggestions from the literature [6, 8, 12, 13, 20].

## Data Availability

The approved study design contained commitment to keep the data material only for the researchers, and hence, unfortunately, the data material cannot be distributed.

## Abbreviations

CI: Confidence interval
DE: Delta efficiency
*E*_0_: Internal energy expenditure in cycling work
*E*_0, *e*_: Extrapolated zero load cycling energy expenditure
*E*_0, *m*_: Measured zero load cycling energy expenditure
*E*_rest_: Resting energy expenditure
*E*_tot_: Total energy expenditure
*E*_tot,Aer_: Total aerobic energy expenditure
*E*_tot,Anaer_: Total anaerobic energy expenditure
GE: Gross efficiency
La: Lactate level (mmol/l)
NE: Net efficiency
*P*max: Maximum power in incremental test
RER: Respiratory exchange ratio
SD: Standard deviation
*T*: Economy of cycling
VO_2_: Oxygen consumption
VO_2max_: Maximal oxygen consumption
WE: Work efficiency
*WE*_*e*_: Extrapolated work efficiency
*WE*_*m*_: Measured work efficiency
*W*_ext_: External work
